# Variability in body weight precedes diagnosis in dementia: A nationwide cohort study

**DOI:** 10.1002/brb3.1811

**Published:** 2020-08-28

**Authors:** Jane Ha, Yeongkeun Kwon, Ye‐Ji Kwon, DaHye Kim, Kyungdo Han, Mi Jang, Sungsoo Park, Ga Eun Nam, Yang Hyun Kim, Do Hoon Kim, Yong Gyu Park, Kyung‐Hwan Cho

**Affiliations:** ^1^ Department of Medicine Korea University College of Medicine Seoul Korea; ^2^ Center for Obesity and Metabolic Diseases Korea University Anam Hospital Seoul Korea; ^3^ Division of Foregut Surgery Korea University College of Medicine Seoul Korea; ^4^ Department of Neurology Korea University College of Medicine Seoul Korea; ^5^ Department of Biostatistics College of Medicine The Catholic University of Korea Seoul Korea; ^6^ Department of Biotechnology and Food Science Norwegian University of Science and Technology Trondheim Norway; ^7^ Department of Family Medicine Korea University College of Medicine Seoul Korea

**Keywords:** Alzheimer's disease, body weight, cohort study, dementia, vascular dementia

## Abstract

**Background:**

While there have been disagreements concerning whether obesity and increase in body weight elevate the risk of dementia, variability in body weight has been recently recognized as a new biometric associated with a high risk for a number of diseases. This nationwide, population‐based cohort study examined the association between body weight variability and dementia.

**Methods:**

A total of 2,812,245 adults (mean age, 51.7 years; standard deviation, 8.6) without a history of dementia who underwent at least three health examinations between 2005 and 2012 in a nationwide cohort were followed‐up until the date of dementia diagnosis (based on prescribed drugs and disease code) or until 2016 (median follow‐up duration, 5.38 years; interquartile range, 5.16–5.61). Cox regression models were used to evaluate the risk of Alzheimer's disease and vascular dementia according to body weight variability.

**Results:**

The hazard ratios (95% confidence intervals) of the highest quartiles of variability were 1.42 (1.35–1.49) for Alzheimer's disease and 1.47 (1.32–1.63) for vascular dementia compared to the lowest quartile group as a reference. This association was consistent in various subgroup analyses and sensitivity analyses.

**Conclusions:**

Body weight variability could predict Alzheimer's disease and vascular dementia, which may provide new insights into the prevention and management of dementia.

## Graphical abstract

Body weight variability could be a potential risk factor for Alzheimer's disease and vascular dementia, which may provide new insights for dementia prevention.

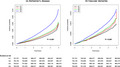

## 1 INTRODUCTION

Dementia plays a substantial role in the mortality of elderly populations (Guehne, Riedel‐Heller, & Angermeyer, 2005) and has a significant impact with regard to costs and resource utilization in healthcare (Wimo, Jönsson, Bond, Prince, & Winblad, 2013). However, in the absence of treatments that block disease progression, better understanding of preventive measures is fundamental for reducing the number of individuals affected by dementia (Alzheimer's Association, (2015)).

Body weight (BWt) is thought to be associated with the risk of dementia. Obesity has been shown to relate to changes in brain structure (Gustafson, Steen, & Skoog, 2004; Haltia, Viljanen, & Parkkola, 2007) and function, (Delgado, Violante, Nieto‐Charques, & Cerdan, 2011) cognitive deficits, (Kilander, Nyman, Boberg, & Lithell, 1997; Sørensen, 1982) and dementia including Alzheimer's disease (AD; Gustafson, 2008) Recent studies have shown that a high body mass index (BMI) increases the risk of dementia over the long term (>20 years) but lowers the risk in the shorter term (<10 years; Kivimäki et al., 2018; Singh‐Manoux et al., 2018) These findings are consistent with a large cohort study with a follow‐up duration of 9.1 years, which showed that being underweight increases the risk of dementia (Qizilbash et al. (2015)). Meanwhile, other studies have concluded that not only weight loss, but also weight gain is associated with a high risk of dementia (Power et al., 2013; Ye, Jang, & Kim, 2016).

Variability in BWt has been recognized as a new biometric that confers an elevated risk of diverse diseases including cardiovascular disease (Field, Byers, & Hunter, 1999; French et al., 1997) and diabetes.(Field et al., 2004; Maruthur, Ma, & Delahanty, 2013) Dementia is also thought to be related to BWt variability; Ravona‐Springer et al. showed that 40‐ to 70‐year‐old individuals with higher BWt variability had a higher risk of dementia after 36 years.(Ravona‐Springer, Schnaider‐Beeri, & Goldbourt, 2013) The present large‐scale cohort study with a 5‐year follow‐up, which included more than 2,810,000 Korean adults, aimed to examine the relationship between BWt variability and the risk of dementia.

## 2 METHODS

### 2.1 Data source and study population

The National Health Insurance Corporation is the single insurer operated by the government covering approximately 97% of the Korean population. Subscribers are recommended to undergo a standardized medical examination at least every two years. Among 23,503,802 individuals who underwent health examinations between 1 January 2009 and 31 December 2012 (index year), those who underwent fewer than three examinations from 2005, had missing data on at least one variable, had a change in height of > 5 cm, or had dementia before the index year were excluded. Individuals with dementia were defined based on the International Classification of Disease, 10th Revision (ICD‐10) codes or the prescription of antidementia drugs. Ultimately, the study population consisted of 2,812,245 individuals (Figure S1). This study was approved by the Institutional Review Board of Korea University Anam Hospital (No. 2018AN0257). The requirement for written informed consent was waived by the committee.

### 2.2 Variability in body weight

Three indices of variability were used to define BWt variability: (a) variability independent of the mean (VIM), (b) standard deviation (*SD*), and (c) coefficient of variation (CV). VIM was calculated as 100 × *SD*/Mean^beta^, where the power beta was derived by nonlinear regression on the basis of the natural logarithm of the *SD* over the natural logarithm of the mean. *SD* is the square root of the mean of the squared differences of the mean and each measurement. CV was calculated as 100 × *SD*/Mean.

### 2.3 Study outcome and follow‐up

Dementia was defined according to two or more prescriptions of antidementia drugs (four classes: rivastigmine, galantamine, memantine, and donepezil hydrochloride) and assigned disease codes for AD (ICD‐10 F00 or G30) and vascular dementia (VaD) (ICD‐10 F01.0, F01.1, F01.2, F01.3, F01.8, or F01.9). Participants were followed‐up from the index year until the date of dementia diagnosis or 31 December 2016, whichever occurred earlier.

### 2.4 Assessments of comorbidities and other variables

The presence of diabetes was defined by the following criteria: (a) one or more claims per year under ICD‐10 codes E10–14 and one or more claims per year for the prescription of antidiabetic medication, or (b) fasting glucose level ≥ 126 mg/dl. The presence of hypertension was defined by the following criteria: (a) one or more claims per year under ICD‐10 codes I10 or I11 and at least one claim per year for the prescription of antihypertensive agents, or (b) systolic/diastolic blood pressure ≥ 140/90 mmHg. The presence of dyslipidemia was defined by the following criteria: (a) at least one claim per year under ICD‐10 code E78 and at least one claim per year for the prescription of a lipid‐lowering agent, or (b) total cholesterol ≥ 240 mg/dl. The presence of stroke or coronary artery disease was recorded based on self‐report questionnaire data, and depression was defined according to ICD‐10 codes F32 or F33.

Waist circumference (WC) was measured during minimal respiration at the narrowest point between the inferior border of the rib cage and the iliac crest. Body mass index (BMI) was calculated as the body weight (BWt) in kilograms divided by square of the height in meters. The data for each participant's smoking status, alcohol consumption, and regular exercise were obtained by a standardized self‐report questionnaire.(Lee, Han, Ko, Ko, & Lee, 2016) Participants were categorized as nonsmokers, ex‐smokers, or current smokers based on their smoking status. Alcohol consumption was recorded based on the participant's frequency of alcohol consumption per week (none; mild, ≤twice/week; heavy, ≥three times/week). Regular exercise was defined depending on whether the participant worked out at least five times per week. Income level was dichotomized at the lower 10%. Serum glucose, cholesterol, liver enzymes, and hemoglobin levels were measured after an overnight fast. Hospitals in which the health examinations occurred were certified and controlled by the NHIS.

### 2.5 Statistical analyses

Baseline characteristics are presented as mean (*SD*) or number of participants (%). Participants were classified into four groups by BWt variability quartiles using VIM, *SD*, and CV. Incidence rate was calculated by the number of incident cases divided by the total follow‐up duration (person years). Kaplan–Meier curves with log‐rank test were used to compare incidence probabilities by quartiles of BWt variability. Hazard ratios (HRs) and 95% confidence intervals (95% CI) for AD and VaD were analyzed using Cox regression analysis for quartile groups of BWt variability. Proportional hazards assumption was assessed by the Schoenfeld residuals test with the logarithm of the cumulative hazard function based on Kaplan–Meier estimations for quartiles of BWt variability. Multivariable‐adjusted proportional hazards models were utilized: Model 1 was adjusted for age and sex; Model 2 was further adjusted for mean BMI, WC, smoking status, alcohol consumption, regular exercise, income, and the presence of hypertension, diabetes, dyslipidemia, stroke, coronary artery disease, or depression; Model 3 was further adjusted for systolic blood pressure and serum levels of glucose during fasting, total cholesterol, hemoglobin, and liver enzymes.

To evaluate whether the effect of BWt variability varies across subgroups, we tested the interaction between group allocations and factor categories with Cox regression analysis, by including an interaction term between the subgroup category and the study group. HR (95% CI) of the highest quartile (Q4) group was measured compared with the lower three quartiles (Q1–Q3) as a reference group in subgroups based on direction of change of the first and the last measured BWt (decreased, sustained, and increased group at 5% change of BWt), age, sex, BMI, WC, smoking status, alcohol consumption, regular exercise, income, and the presence of hypertension, diabetes, dyslipidemia, stroke, coronary artery disease, or depression.

To prevent the possible effects of multicollinearity caused by the association between VIM and the mean BMI, we also used the baseline BMI level instead of the mean BMI level in the sensitivity analysis. Sensitivity analyses were performed excluding those individuals with: (a) dementia occurring within 2 years of follow‐up, (b) coronary artery disease or stroke, or (c) hypertension, diabetes, or dyslipidemia.

Statistical analyses were performed using SAS version 9.4 (SAS Institute Inc.), and a 2‐tailed *p* value < .05 was considered statistically significant.

## 3 RESULTS

Participant characteristics, arranged by quartiles of BWt variability measured by VIM, are shown in Table 1. The mean age of the included participants at baseline was 51.7 (8.6) years. Individuals in higher quartiles of BWt variability were more likely to be female and tended to have a lower BMI and WC compared with individuals in lower quartiles. For trend values, *p* was < .001 for all variables.

**Table 1:** Baseline characteristics of the study population

Variables	Body weight categories according to variability			
	Q1 (*n* = 701,701)	Q2 (*n* = 704,145)	Q3 (*n* = 703,021)	Q4 (*n* = 703,378)
Age, mean (*SD*), y	51.6 (8.3)	51.5 (8.3)	51.5 (8.5)	52.0 (9.2)
Female sex, *N* (%)	206,259 (29.4)	223,643 (31.8)	230,026 (32.7)	272,969 (38.8)
BMI, mean (*SD*), kg/m^2^	24.1 (2.9)	24.0 (2.9)	24.0 (2.9)	23.9 (3.2)
WC, mean (*SD*), cm	82.0 (8.3)	81.5 (8.3)	81.4 (8.3)	81.0 (8.7)
Systolic BP, mean (*SD*), mm Hg	123.4 (14.0)	123.2 (14.0)	123.2 (14.1)	123.1 (14.4)
Diastolic BP, mean (*SD*), mm Hg	77.5 (9.7)	77.4 (9.7)	77.3 (9.7)	77.1 (9.8)
Smoking status				
Never smoked, *N* (%)	345,361 (49.3)	354,331 (50.4)	354,539 (50.5)	379,497 (54.0)
Former smoker, *N* (%)	168,201 (24.0)	160,311 (22.8)	158,022 (22.5)	147,299 (21.0)
Current smoker, *N* (%)	187,579 (26.8)	188,950 (26.9)	189,983 (27.0)	176,148 (25.1)
Alcohol consumption^a^				
Nonuser, *N* (%)	302,166 (43.1)	312,476 (44.5)	319,433 (45.5)	353,833 (50.4)
Mild drinker, *N* (%)	339,283 (48.4)	334,637 (47.6)	327,622 (46.7)	299,665 (42.7)
Heavy drinker, *N* (%)	58,931 (8.4)	55,761 (7.9)	54,794 (7.8)	48,824 (7.0)
Regular exercise^b^, *N* (%)	168,872 (24.1)	168,353 (23.9)	166,506 (23.7)	162,048 (23.1)
Low income^c^, *N* (%)	135,399 (19.3)	142,766 (20.3)	147,560 (21.0)	165,436 (23.5)
Comorbidities				
Hypertension, *N* (%)	206,661(29.5)	203,721(28.9)	204,092 (29.0)	211,936 (30.1)
Diabetes, *N* (%)	70,817 (10.1)	70,396 (10.0)	73,555 (10.5)	83,880 (11.9)
Dyslipidemia, *N* (%)	160,244 (22.8)	159,039 (22.6)	159,995 (22.8)	165,264 (23.5)
Stroke, *N* (%)	3,076 (0.6)	3,089 (0.6)	3,464 (0.7)	4,674 (1.0)
Coronary artery disease, *N* (%)	10,638 (2.1)	10,277 (2.0)	11,035 (2.2)	12,884 (2.7)
Depression, *N* (%)	19,947 (2.8)	21,506 (3.1)	23,805 (3.4)	32,963 (4.7)
Laboratory results				
Fasting plasma glucose, mean (*SD*), mg/dL	99.6 (22.1)	99.3 (22.2)	99.5 (23.2)	100.1 (25.9)
Total cholesterol, mean (*SD*), mg/dL	198.3 (35.4)	198.0 (35.5)	197.8 (35.8)	197.0 (36.7)
HDL cholesterol, mean (*SD*), mg/dL	53.7 (15.3)	54.3 (15.4)	54.5 (15.4)	55.3 (15.7)
LDL cholesterol, mean (*SD*), mg/dL	117.0 (35.2)	116.7 (35.2)	116.4 (35.6)	115.5 (35.6)
Triglyceride, median (IQR), mg/dL	123.4 (123.3–123.6)	120.8 (120.6–121.0)	120.0 (119.8–120.1)	115.7 (115.6–115.9)
Hemoglobin, mean (*SD*), g/dL	14.4 (1.5)	14.3 (1.5)	14.3 (1.5)	14.1 (1.6)
AST, median (IQR), IU/L	25.3 (25.3–25.4)	25.3 (25.2–25.3)	25.3 (25.3–25.3)	25.3 (25.3–25.3)
ALT, median (IQR), IU/L	23.7 (23.6–23.7)	23.3 (23.3–23.3)	23.2 (23.2–23.3)	22.8 (22.8–22.9)

Abbreviations: ALT, alanine aminotransferase; AST, aspartate aminotransferase; BMI, body mass index; BP, blood pressure; HDL, high‐density lipoprotein; IQR, interquartile range; LDL, low‐density lipoprotein; WC, waist circumference.

Variability was calculated using VIM (variability independent of mean), and subjects were classified into four groups according to the variability quartiles.

^a^ Alcohol consumption was categorized based on the frequency of alcohol consumption per week [none; mild, ≤ twice/week; heavy, ≥ three times/week].

^b^ Regular exercise was defined as physical activity that was performed at least five times per week.

^c^ Income level was dichotomized at the lower 10%.

**Figure 1:**
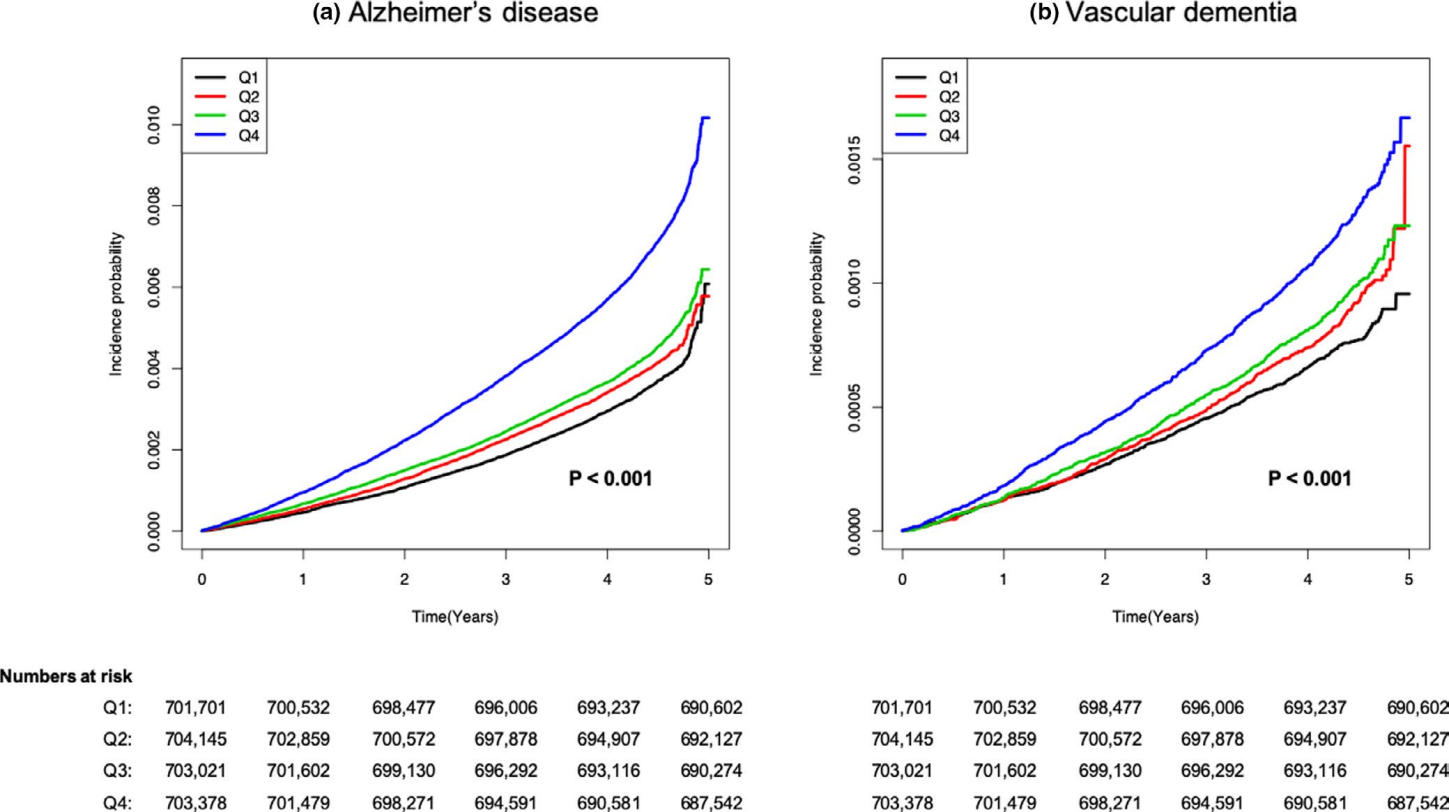
Kaplan–Meier curves of cumulative probability of dementia incidence. Incidence probabilities of (a) Alzheimer's disease and (b) vascular dementia, stratified by body weight variability. Q1 (low variability) to Q4 (high variability) are quartiles of variability in body mass index. A significant difference in risk for each type of dementia was seen between the groups (log‐rank *p* < .001)

There were 9,262 cases of AD (0.33%) and 1,894 cases of VaD (0.07%) during the follow‐up period (median [interquartile range], 5.38 [5.16–5.61] years). Incrementally higher risk outcomes were observed with higher VIM quartiles, compared to lower quartile groups (Figure 1). Individuals in the Q4 group had an approximately 42% higher risk of AD and 46% higher risk of VaD compared with those in the Q1 group (Table 2). Table S1 shows that HRs for the risk of dementia were higher in the Q4 group compared to the Q1 group in each subgroup divided by the baseline BMI and the direction of BWt change, respectively, except subgroups with BMI ≥ 30 kg/m^2^ and BMI < 18.5 kg/m^2^. The results were consistent with analyses using *SD* and CV as measures of BWt variability (Table S2).

**Table 2:** Hazard ratios (95% confidence intervals) for the risk of dementia by quartiles of body weight variability

Categories	*N*	Event	Duration	Incidence rate (per 1,000 person years)	Hazard ratio (95% confidence interval)		
					MODEL1^a^	MODEL2^b^	MODEL3^c^
**Alzheimer's disease**							
Q1	701,701	2,415	3,071,010.39	0.79	1 [Reference]	1 [Reference]	1 [Reference]
Q2	704,145	2,503	3,080,856.90	0.81	1.05 (0.99–1.11)	1.05 (0.99–1.11)	1.05 (0.99–1.11)
Q3	703,021	3,006	3,071,416.67	0.98	1.19 (1.12–1.24)	1.17 (1.11–1.24)	1.16 (1.10–1.22)
Q4	703,378	5,238	3,056,935.52	1.71	1.49 (1.42–1.57)	1.47 (1.40–1.55)	1.42 (1.34–1.49)
**Vascular dementia**							
Q1	701,701	527	3,071,010.39	0.17	1 [Reference]	1 [Reference]	1 [Reference]
Q2	704,145	534	3,080,856.90	0.17	1.02 (0.91–1.15)	1.02 (0.90–1.15)	1.02 (0.90–1.15)
Q3	703,021	649	3,071,416.67	0.21	1.21 (1.08–1.36)	1.20 (1.07–1.34)	1.19 (1.06–1.33)
Q4	703,378	984	3,056,935.52	0.32	1.54 (1.39–1.71)	1.51 (1.36–1.68)	1.47 (1.32–1.63)

Abbreviations: ALT, alanine aminotransferase; AST, aspartate aminotransferase; BMI, body mass index; BP, blood pressure; WC, waist circumference.

Variability was calculated using VIM (variability independent of mean), and subjects were classified into four groups according to the variability quartiles.

^a^ Adjusted for age and female sex.

^b^ Adjusted for age, female sex, mean BMI, WC, smoking status, alcohol consumption, regular exercise, low income, and the presence of hypertension, diabetes, dyslipidemia, stroke, coronary artery disease, or depression.

^c^ Adjusted for age, female sex, mean BMI, WC, smoking status, alcohol consumption, regular exercise, low income, and the presence of hypertension, diabetes, dyslipidemia, stroke, coronary artery disease, or depression; and systolic BP, serum fasting plasma glucose, total cholesterol, hemoglobin, AST, and ALT level.

In every subgroup, the risk of AD increased in individuals with variable BWt, compared with the stable BWt group (Table 3). Significantly higher adjusted HRs of AD were observed among subgroups with the following characteristics: younger age, male, current smoker, heavy drinker, and the presence of depression. The effect of BWt variability on VaD did not show significant interactions between the study groups and each variable. Baseline BMI and direction of body weight change did not affect the adjusted HRs of dementia.

**Table 3:** Adjusted hazard ratios for Alzheimer's disease and vascular dementia based on subgroups in the highest quartile group of body weight variability compared with the lower three quartiles

Variables	Subgroups	Alzheimer's disease		Vascular dementia	
		HR (95% CI)	*p* for interaction	HR (95% CI)	*p* for interaction
Direction of body weight change^a^	Decreased	1.32 (1.20–1.46)	.55	1.29 (1.03–1.62)	.80
	Sustained	1.25 (1.17–1.32)		1.22 (1.06–1.40)	
	Increased	1.22 (1.09–1.36)		1.36 (1.08–1.70)	
Age	< 65 years	1.55 (1.45–1.67)	<.001	1.38 (1.22–1.55)	.85
	≥ 65 years	1.29 (1.24–1.34)		1.37 (1.23–1.53)	
Sex	Male	1.37 (1.31–1.45)	.01	1.42 (1.28–1.57)	.34
	Female	1.28 (1.22–1.35)		1.28 (1.12–1.46)	
Baseline BMI	< 25 kg/m^2^	1.32 (1.26–1.37)	.80	1.41 (1.28–1.55)	.22
	≥ 25 kg/m^2^	1.32 (1.24–1.41)		1.26 (1.10–1.46)	
WC	Male, <90 cm; Female, <85 cm	1.33 (1.27–1.39)	.21	1.32 (1.20–1.45)	.33
	Male, ≥ 90 cm; Female, ≥ 85 cm	1.29 (1.21–1.38)		1.47 (1.26–1.70)	
Smoking status^b^	Never smoked or former smoker	1.31 (1.26–1.36)	.003	1.37 (1.25–1.50)	.80
	Current smoker	1.44 (1.32–1.58)		1.35 (1.15–1.59)	
Alcohol consumption^c^	Nonuser or mild drinker	1.32 (1.27–1.37)	.03	1.37 (1.27–1.49)	.85
	Heavy drinker	1.46 (1.24–1.73)		1.30 (0.94–1.79)	
Regular exercise^d^	No	1.33 (1.27–1.38)	.64	1.41 (1.29–1.54)	.19
	Yes	1.34 (1.23–1.46)		1.23 (1.03–1.48)	
Income^e^	High	1.37 (1.29–1.46)	.61	1.31 (1.13–1.51)	.34
	Low	1.31 (1.25–1.36)		1.40 (1.27–1.54)	
Hypertension	No	1.33 (1.26–1.40)	.07	1.40 (1.23–1.59)	.49
	Yes	1.33 (1.27–1.39)		1.35 (1.22–1.50)	
Diabetes	No	1.35 (1.30–1.41)	.08	1.40 (1.28–1.54)	.43
Diabetes	Yes	1.25 (1.17–1.35)	.08	1.28 (1.10–1.49)	.43
Dyslipidemia	No	1.31 (1.25–1.37)	.79	1.41 (1.27–1.55)	.36
	Yes	1.36 (1.28–1.44)		1.31 (1.15–1.50)	
Stroke	No	1.31 (1.25–1.38)	.07	1.28 (1.15–1.43)	.49
	Yes	1.27 (1.06–1.52)		1.29 (0.94–1.76)	
Coronary artery disease	No	1.33 (1.26–1.40)	.06	1.32 (1.18–1.47)	.57
	Yes	1.21 (1.06–1.38)		1.25 (0.91–1.71)	
Depression	No	1.29 (1.24–1.34)	.04	1.35 (1.24–1.48)	.36
	Yes	1.31 (1.20–1.42)		1.26 (1.02–1.54)	

Abbreviations: BMI, body mass index; CI, confidence interval; HR, hazard ratio; WC, waist circumference.

Variability was calculated using VIM (variability independent of mean), and subjects were classified into four groups according to the variability quartiles.

^a^ Categories of the direction of body weight changes were defined as follows: decreased (5% decrease or more between first and last measurement), sustained (less than 5% increase or decrease between first and last measurement), or increased (5% increase or more between first and last measurement).

^c^ Subjects were classified as Non or mild drinkers (≤ twice/week) or heavy drinkers (≥ three times/week) based on frequency of alcohol consumption per week.

^d^ Regular exercise was defined as physical activity that was performed at least five times per week.

^e^ Income level was dichotomized at the lower 10%.

HRs were nearly identical when the baseline BMI level was used rather than the mean BMI level in Cox regression analysis (Table S3). Excluding individuals with coronary heart disease or stroke, or with hypertension, diabetes, or dyslipidemia did not weaken the association between BWt variability and dementia. Additionally, similar results were obtained after excluding individuals diagnosed with dementia within 2 years of follow‐up.

## 4 DISCUSSION

To our knowledge, this is the first study in a general Asian population with a well‐established longitudinal national database, demonstrating that variable BWt precedes dementia.

A previous study examined the association of BWt variability during midlife and the risk of dementia in men.(Ravona‐Springer et al., (2013)) When the groups were stratified by *SD* quartiles of BWt change among three measurements in five years, the odds ratio for dementia after 36 years was 1.74 (95% CI, 1.14–2.64) in the highest *SD* quartile of BWt change compared to the lowest quartile, and no significant trend was observed across the direction of BWt change. The report supported the significance of BWt variability as a predictor for dementia, whereas the effectiveness of a one‐point measurement of BWt was considered to be controversial considering that an underweight state as well as obesity in midlife increases the risk of dementia (Whitmer, Gunderson, Quesenberry, Zhou, and Yaffe (2007); Kivipelto et al., 2005; Albanese, Launer, & Egger, 2017). The findings of the report are different from those of our study for the interval between weight measurement and assessment of dementia, but they support the significance of BWt variability as a predictor for dementia. The importance of the current findings lies in the observation of a relatively short time period before the onset of dementia, investigating the risk of two types of dementia separately, and considering abundant covariates when providing HRs for each subgroup.

The association between BWt and dementia is thought to be based on different pathological processes at different time points. Specifically, an underweight state and loss of body mass near dementia onset, potential risk factors for dementia (Besser, Gill, & Monsell, 2014; Buchman et al., 2005; Johnson, Wilkins, & Morris, 2006), might reflect preclinical dementia.(Pedditizi, Peters, & Beckett, 2016) While the reasons for the observed association are unclear at present, there would be possible bidirectional interactions between BWt variability and development of dementia. Since the development of dementia considerably affects BWt changes,(Guerin et al., 2005; White, Pieper, & Schmader, 1998) we excluded participants who had dementia within 2 years of follow‐up in order to reduce the effects of reverse causality and still observed increased risk of dementia in individuals with high BWt variability (Table S3). Even though possible reverse causality could not be perfectly excluded, this finding implies significant impacts of BWt variability on dementia. Further, an increased risk of dementia was consistently related to higher BWt variability in each subgroup divided by the direction of BWt change (Table 3). Our results imply that BWt variability, compared to one‐point BWt status or BWt change between two time points, is a potent predictor for developing dementia.

Although our results indicate that variable BWt increases the risk of dementia, they do not show whether an obese or underweight status should be maintained for the prevention of dementia. However, established risk factors for dementia, such as hypertension and diabetes, need to be controlled through the maintenance of normal weight in the elderly.(Dattilo and Kris‐Etherton, (1992); Wing et al., 1987; Anderson & Konz, 2001) During weight management, we suggest that healthcare providers recommend an accurate amount of weight loss to patients and continuously check aspects of BWt changes in order to minimize variability in BWt, since diverse sensitivity analyses, taking into account the effects of comorbidities, showed similar associations between BWt variability and the risk of dementia (Table S3).

We analyzed the risk of AD and VaD differentially. Subgroup analyses showed that unlike in the case of AD, effects of BWt variability on the risk of VaD did not differ according to age, sex, smoking, alcohol consumption, or the presence of hypertension, stroke, coronary artery disease, or depression (Table 3). While studies on the effects of BWt status on VaD are few, varying associations with BWt status when compared to AD have been reported.(Fitzpatrick, Kuller, & Lopez, 2009; Hughes, Borenstein, Schofield, Wu, & Larson, 2009) Further studies focusing on the features of different types of dementia are required for the development of guidelines for weight control in patients at risk of each type of dementia.

Some limitations in our study should be acknowledged. First, inconsistencies between the diagnosis of patients in medical practice and claim data records may have caused inaccurate analyses. For example, the exclusion of subjects diagnosed with dementia in prior to the index year might have led to the exclusion of subjects with unrecorded dementia. However, the probability of having undiagnosed dementia is thought to be very low because public medical service is readily accessible in Korea and each local government provides free screening for cognitive function for the elderly. Second, the homogeneity of the study population may present an issue. As only Koreans were included in the study, extrapolation to individuals of different ethnicities should be undertaken with caution. Moreover, we selected subjects aged 40 years or older who underwent health examinations at least 3 times in order to assess the body weight variability. As such, the study population was skewed toward young subjects. Third, because of the limitations of claim data, there were some variables with limited information (classification of smoking status, alcohol consumption, and exercise based on questionnaires) and inaccessible variables, which might have resulted in the possibility of residual confounds such as intention of BWt change (Wannamethee, Shaper, Whincup, & Walker, 2000), presence of the apolipoprotein E ϵ4 allele (APOE ϵ4; Evans, Beckett, & Field, 1997), and level of education (Stern et al., (1994)); further studies examining these risk factors are therefore required. Fourth, the competing risk of death was not considered in this study. Fifth, due to the large size of the cohort, there is a risk of statistical false positives.

## 5 CONCLUSION

In this study of a nationally representative cohort, we observed a dose–response relationship between BWt variability and the risk of AD and VaD. The associations were consistent among various subgroups and sensitivity analyses. Future studies should investigate mechanisms underlying the interaction between BWt variability and dementia, and further elaborate on the prevention and management of dementia regarding BWt variability.

## DISCLOSURES

The authors declare no financial or other conflicts of interest.

## AUTHORS CONTRIBUTION

J.H. interpreted results and wrote the manuscript. Y.K. designed the study, performed data analysis, and wrote the manuscript. Y‐J.K. interpreted results and edited the manuscript. D.K and K.H. conducted statistical analyses. M.J. and S.P. critically revised the manuscript. G.E.N. collected and analyzed data. Y.H.K. and D.H.K. critically revised the manuscript. Y.G.P. collected data and supervised the analyses. K‐H.C. supervised this work. All authors discussed the results and approved the final manuscript.

### Peer Review

The peer review history for this article is available at https://publons.com/publon/10.1002/brb3.1811.

## Supporting information

Supplementary Material

## Data Availability

Data are available from the National Health Insurance Service Institutional Data Access for researchers who meet the access criteria for confidential data. All analyzed data are included in this article and its online supplemental materials.
