# Supplementary material for: Variability in body weight precedes diagnosis in dementia: A nationwide cohort study
Source: Brain Behav. 2020 Aug 28;10(11):e01811. doi: 10.1002/brb3.1811 (PMC7667348; doi:10.1002/brb3.1811)
Supplement: Supplementary file 1 — Supplementary Material [file BRB3-10-e01811-s001.docx]

**Figure S1.** Flow chart of the study population


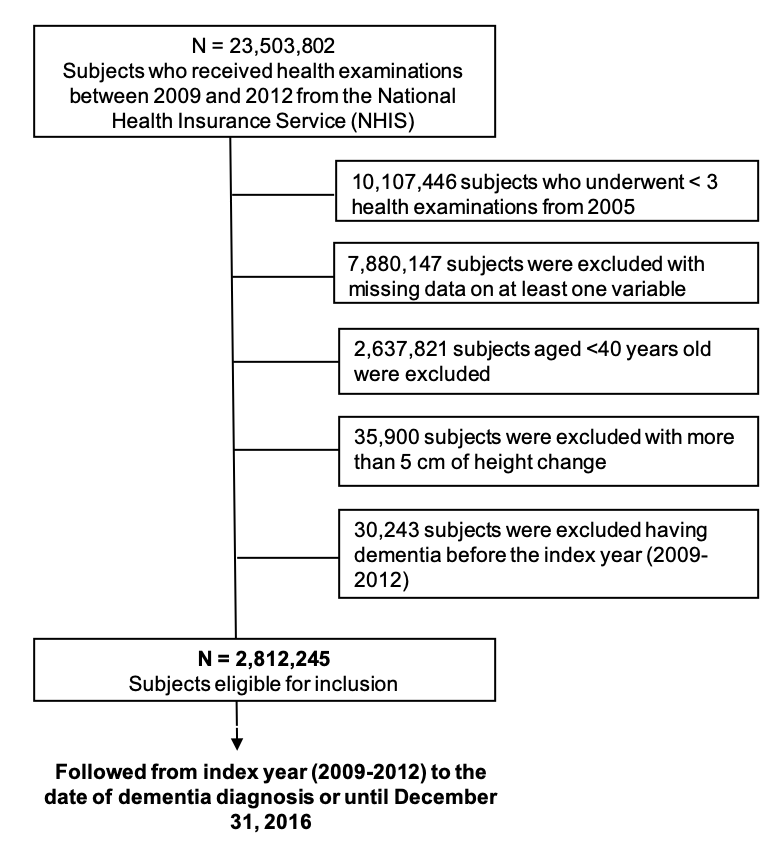


| **Table S1. HRs (95% CI) for the risk of dementia according to baseline BMI and body weight variability** | | | | | |
| --- | --- | --- | --- | --- | --- |
| **Categories according to body weight variability** | **BMI categories according to baseline BMI (kg/m^2^)** | | | | |
|  | **<18.5**  (N=71,544) | **18.5–23**  (N=984,656) | **23–25**  (N=724,015) | **25–30**  (N=944,631) | **>30**  (N=87,399) |
| **Alzheimer’s disease** | | | | | |
| **Q1** | 1 [Reference] | 1 [Reference] | 1 [Reference] | 1 [Reference] | 1 [Reference] |
| **Q2** | 1.02 (0.71–1.47) | 1.02 (0.93–1.12) | 1.18 (1.06–1.31) | 0.97 (0.88–1.08) | 0.89 (0.62–1.28) |
| **Q3** | 1.05 (0.77–1.45) | 1.10 (1.00–1.20) | 1.27 (1.15–1.42) | 1.15 (1.04–1.26) | 1.17 (0.84–1.63) |
| **Q4** | 1.28 (0.97–1.68) | 1.32 (1.22–1.43) | 1.56 (1.41–1.72) | 1.46 (1.33–1.60) | 1.26 (0.93–1.71) |
| **Vascular dementia** | | | | | |
| **Q1** | 1 [Reference] | 1 [Reference] | 1 [Reference] | 1 [Reference] | 1 [Reference] |
| **Q2** | 1.84 (0.71–4.75) | 0.95 (0.77–1.17) | 1.03 (0.81–1.31) | 1.06 (0.87–1.29) | 0.70 (0.32–1.54) |
| **Q3** | 2.98 (1.29–6.86) | 1.12 (0.92–1.36) | 1.15 (0.92–1.45) | 1.24 (1.03–1.50) | 0.73 (0.34–1.57) |
| **Q4** | 2.82 (1.26–6.30) | 1.44 (1.20–1.73) | 1.66 (1.34–2.05) | 1.32 (1.09–1.59) | 0.94 (0.48–1.84) |
| Abbreviations: BMI, body mass index; HR, hazard ratio; CI, confidence interval.  Variability was calculated using VIM (variability independent of mean), and subjects were classified into four groups based on the variability quartiles. | | | | | |

| **Table S2. HRs (95% CI) for the risk of dementia according to quartiles of body weight variability: the results using SD and CV as indices of variability.** | | | | | | | |
| --- | --- | --- | --- | --- | --- | --- | --- |
| **Categories** | **N** | **Event** | **Duration** | **Incidence rate (per 1,000 person years)** | **HR (95% CI)** | | |
|  |  |  |  |  | **MODEL 1^a^** | **MODEL 2^b^** | **MODEL 3^c^** |
| **Alzheimer’s disease: Analyses using SD as variability index** | | | | | | | |
| **Q1** | 718,018 | 2,435 | 3,146,087 | 0.77 | 1 [Reference] | 1 [Reference] | 1 [Reference] |
| **Q2** | 732,121 | 2,854 | 3,203,328 | 0.89 | 1.09 (1.03–1.15) | 1.09 (1.03–1.15) | 1.09 (1.03–1.15) |
| **Q3** | 656,174 | 3,652 | 2,862,843 | 1.28 | 1.26 (1.20–1.33) | 1.25 (1.19–1.32) | 1.25 (1.19–1.31) |
| **Q4** | 705,932 | 4,221 | 3,067,961 | 1.38 | 1.49 (1.41–1.56) | 1.47 (1.40–1.54) | 1.45 (1.38–1.52) |
| **Alzheimer’s disease: Analyses using CV as variability index** | | | | | | | |
| **Q1** | 704,634 | 2,406 | 3,083,803 | 0.78 | 1 [Reference] | 1 [Reference] | 1 [Reference] |
| **Q2** | 701,611 | 2,446 | 3,069,942 | 0.80 | 1.05 (0.99–1.11) | 1.04 (0.99–1.11) | 1.04 (0.98–1.10) |
| **Q3** | 702,681 | 3,028 | 3,069,735 | 0.99 | 1.19 (1.13–1.25) | 1.18 (1.12–1.25) | 1.17 (1.11–1.23) |
| **Q4** | 703,319 | 5,282 | 3,056,740 | 1.73 | 1.50 (1.43–1.57) | 1.48 (1.41–1.55) | 1.42 (1.36–1.50) |
| **Vascular dementia: Analyses using SD as variability index** | | | | | | | |
| **Q1** | 718,018 | 514 | 3,146,087 | 0.16 | 1 [Reference] | 1 [Reference] | 1 [Reference] |
| **Q2** | 732,121 | 608 | 3,203,328 | 0.19 | 1.12 (1.00–1.26) | 1.12 (0.99–1.26) | 1.11 (0.99–1.25) |
| **Q3** | 656,174 | 679 | 2,862,843 | 0.24 | 1.24 (1.10–1.39) | 1.23 (1.09–1.38) | 1.21 (1.08–1.36) |
| **Q4** | 705,932 | 893 | 3,067,961 | 0.29 | 1.62 (1.45–1.81) | 1.60 (1.44–1.79) | 1.54 (1.38–1.72) |
| **Vascular dementia: Analyses using CV as variability index** | | | | | | | |
| **Q1** | 704,634 | 526 | 3,083,803 | 0.17 | 1 [Reference] | 1 [Reference] | 1 [Reference] |
| **Q2** | 701,611 | 535 | 3,069,942 | 0.17 | 1.04 (0.92–1.17) | 1.03 (0.92–1.17) | 1.03 (0.91–1.16) |
| **Q3** | 702,681 | 643 | 3,069,735 | 0.21 | 1.20 (1.07–1.35) | 1.19 (1.06–1.34) | 1.18 (1.05–1.33) |
| **Q4** | 703,319 | 990 | 3,056,740 | 0.32 | 1.55 (1.39–1.72) | 1.52 (1.36–1.69) | 1.47 (1.32–1.64) |
| Abbreviations: HR, hazard ratio; CI, confidence interval; SD, standard deviation; CV, coefficient of variance.  Variability was calculated using VIM (variability independent of mean), and individuals were classified into four groups based on the variability quartiles.  **^a^** Adjusted for age and sex.  **^b^** Adjusted for age, sex, mean body mass index, waist circumference, smoking status, alcohol consumption, regular exercise, income, and the presence of hypertension, diabetes, dyslipidemia, stroke, coronary artery disease, or depression.  **^c^** Adjusted for age, sex, mean body mass index, waist circumference, smoking status, alcohol consumption, regular exercise, income, and the presence of hypertension, diabetes, dyslipidemia, stroke, coronary artery disease, or depression; and systolic blood pressure and serum levels of fasting plasma glucose, total cholesterol, hemoglobin, aspartate aminotransferase and alanine aminotransferase. | | | | | | | |

| **Table S3. Sensitivity analyses.** | | |
| --- | --- | --- |
| **Various settings in which sensitivity analyses were performed** | **HR (95% CI)** | |
|  | **Alzheimer’s disease** | **Vascular dementia** |
| **Using Cox proportional hazards model incorporating baseline BMI instead of mean BMI** |  |  |
| **Q1** | 1 [Reference] | 1 [Reference] |
| **Q2** | 1.06 (1.01–1.12) | 1.07 (0.98–1.17) |
| **Q3** | 1.20 (1.14–1.26) | 1.15 (1.05–1.26) |
| **Q4** | 1.48 (1.42–1.55) | 1.44 (1.33–1.57) |
| **Excluding individuals with coronary heart disease or stroke** |  |  |
| **Q1** | 1 [Reference] | 1 [Reference] |
| **Q2** | 1.04 (0.99–1.10) | 1.05 (0.99–1.11) |
| **Q3** | 1.16 (1.10–1.21) | 1.16 (1.10–1.22) |
| **Q4** | 1.47 (1.40–1.53) | 1.42 (1.35–1.49) |
| **Excluding individuals with hypertension, diabetes, or dyslipidemia** |  |  |
| **Q1** | 1 [Reference] | 1 [Reference] |
| **Q2** | 1.00 (0.89–1.11) | 1.00 (0.94–1.07) |
| **Q3** | 1.14 (1.03–1.26) | 1.14 (1.07–1.22) |
| **Q4** | 1.37 (1.24–1.50) | 1.38 (1.30–1.46) |
| **Excluding individuals who developed dementia within 2 years of follow-up** |  |  |
| **Q1** | 1 [Reference] | 1 [Reference] |
| **Q2** | 1.02 (0.90–1.15) | 1.05 (0.92–1.19) |
| **Q3** | 1.19 (1.06–1.33) | 1.25 (1.10–1.41) |
| **Q4** | 1.47 (1.32–1.63) | 1.50 (1.34–1.68) |
| Abbreviations: BMI, body mass index; HR, hazard ratio; CI, confidence interval.  Variability was calculated using VIM (variability independent of mean), and individuals were classified into four groups based on the variability quartiles. | | |
